# Human monkeypox: a comparison of the characteristics of the new epidemic to the endemic disease

**DOI:** 10.1186/s12879-022-07900-7

**Published:** 2022-12-12

**Authors:** Sharon Sukhdeo, Sharmistha Mishra, Sharon Walmsley

**Affiliations:** 1 Department of Medicine, University of Toronto, Toronto, Canada; 2 Division of Infectious Diseases, Department of Medicine, St. Michael’s Hospital, MAP Centre for Urban Health Solutions, University of Toronto, Toronto, Canada; 3 Department of Medicine, Division of Infectious Diseases, University Health Network, University of Toronto, Toronto, Canada

**Keywords:** Human monkeypox, Orthopoxvirus, Narrative review, Tecovirimat, Brincidofovir, Vaccine

## Abstract

In May 2022, a new global outbreak of mpox (formerly, human monkeypox) emerged that was declared a public health emergency of international concern by the World Health Organization on July 23, 2022. With new patterns of person-to-person spread within sexual networks in nonendemic countries and several differences from the classic disease course, we performed a comprehensive review of existing literature on human monkeypox to discuss epidemiology, modes of transmission, clinical presentation and asymptomatic infection, diagnostics, therapeutics, and vaccines with the primary aim to identify important areas for future research of this new epidemic form of the disease. A comprehensive literature search was performed of all published literature to August 15, 2022. Historically, in regions of monkeypox virus endemicity, human outbreaks have occurred related to discrete zoonotic events. The animal reservoir is unknown, but the virus has been isolated from rodents. Traditionally, transmission occurred by direct or indirect contact with an infected animal. In nonendemic countries affected in the 2022 outbreak, almost exclusive person-to-person spread has been observed, and most cases are connected to sexual networks of gay, bisexual, and other men who have sex with men. After an incubation period of approximately 13 days, in traditional human cases affected persons developed a febrile prodrome preceding a rash that started on the face and body, spread centrifugally to the palms and soles and healed monomorphically over two to four weeks. However, in the 2022 outbreak, the febrile illness is often absent or occurs after the onset of the rash. The rash presents primarily in the anogenital region and face before disseminating throughout the body, with lesions displaying regional pleomorphism. There is a paucity of data for the role of antiviral agents or vaccines. The epidemiology and clinical course of mpox has changed in the 2022 epidemic from that observed with the endemic disease. There is an urgent need to establish rapid and collaborative research platforms to diagnose, treat and prevent disease and inform important public health and other strategies to stop the spread of disease.

## Background

Mpox (formerly human monkeypox) is a zoonotic disease caused by the monkeypox virus (MPXV), an enveloped, double-stranded DNA virus of the *Orthopoxvirus* genus of the *Poxviridae* family. This genus also includes variola virus (the cause of smallpox), vaccinia virus (from which the smallpox vaccine was derived), and cowpox virus. Three phylogenetically distinct clades of MPXV have been recognized to date. There has been a call for an important paradigm shift in the scientific nomenclature to reflect neutral, descriptive accuracy and de-stigmatization of the virus. Until a change is officially declared by the International Committee on Taxonomy of Viruses, we herein refer to these clades numerically in order of discovery and in line with that suggested by Happi et al.: To this end, “Central African” or “Congo Basin” clade will be referred to as clade I, and the “West(ern) African” clade as clades II and III [1]. Based on limited ecological surveys in select countries within Africa and Asia in the 1970–1980 s, clade I is endemic to the Democratic Republic of the Congo (DRC), Cameroon, the Central African Republic, Gabon, and the Republic of the Congo. By similar methods, clade II is endemic to Nigeria, Sierra Leone, Liberia, the Ivory Coast, and Ghana. Clade III refers to most sequences identified in the 2017, 2018, and 2022 outbreaks discussed below [1]. An animal reservoir of MPXV has not been identified; however, MPXV has been identified in rodents such as giant pouched rats and squirrels [2–4]. Humans and non-human primates are not required for the viral life cycle and thus are only incidental hosts.

In May 2022, a new global outbreak of mpox emerged in previously non-endemic countries that was subsequently declared a public health emergency of international concern by the World Health Organization (WHO) on July 23, 2022. Given several differences from the classic disease course, this outbreak called for a rapid review of existing literature to highlight important areas of poor understanding to inform future research. We provide a comprehensive temporal review of mpox to compare to the endemic disease and discuss relevant issues in the current epidemic with respect to epidemiology, modes of transmission, clinical presentation and asymptomatic infection, diagnostics, therapeutics, and vaccines. We aim to outline several areas in need of new or updated research that should be incorporated into globally collaborative research platforms with the common goal of preventing further morbidity and mortality.

## Methods

We performed two comprehensive English language published literature searches using six online databases (Cochrane Library, EMBASE, LILACS, PubMed, Science Direct, and Web of Science) on June 16, 2022 and updated it on August 15, 2022 to yield additional literature relevant to the 2022 outbreak. Primary search terms included “monkeypox”, “monkeypox virus”, or “Orthopoxvirus”. Secondary search terms were used to accompany each primary search term, and included “transmission”, “communicability”, “public health”, “zoonosis”, “zoonotic”, “droplet”, “airborne”, “aerosol”, “sexual”, “incubation period”, “clinical characteristics”, “complications”, “severe”, “severe disease”, “asymptomatic”, “presymptomatic”, “subclinical”, “paucisymptomatic”, “clinically inapparent”, “diagnosis”, “treatment”, “tecovirimat”, “ST-246”, “brincidofovir”, “CMX001”, “CMX 001”, “hexadecyloxypropyl-cidofovir”, “HDP-cidofovir”, “vaccine”, “infection prevention”, and “healthcare workers”. An additional review of the references of retrieved literature was then performed. To capture newly published guidelines and interim recommendations, additional searches of websites were done including WHO.int, CDC.gov, and Canada.ca.

## Results

### Epidemiologic review

#### Denmark, 1958

In 1958, two separate outbreaks of a pox-like disease occurred in Macaca cynomolgus monkeys in Copenhagen, Denmark [5]. Starting as early as 51 days after arrival from Singapore, 20% of monkeys in the first outbreak and 30% in the second outbreak developed clinical disease. The virus isolated from dermal lesions of affected monkeys was similar to but distinct from other poxviruses and was thus recognized as the newest member of the poxvirus group and designated “monkeypox virus”. Acknowledging the apparently long incubation period, researchers hypothesized that several cycles of inapparent infection preceded identifiable disease. It was unknown whether MPXV was introduced by a monkey with recent infection (such as through nasopharyngeal colonization) or a more remote infection (such as through latent carrier in tissues) [6]. The lesions were likely pruritic (deduced from self-incurred excoriations), and lesions were seen to be the largest, most abundant, and often umbilicated on the palms and soles [5]. Most of the lesions appeared to be in the same stage of eruption with occasional variations. In the convalescent stage, scars were readily identifiable. There were no deaths attributed to the disease. On autopsies of monkeys with clinical disease, no lesions were observed in any organs. However, MPXV was isolated from kidneys of otherwise apparently healthy monkeys euthanized for other purposes raising the issue of possible asymptomatic infection. Subsequent animal studies by von Magnus et al. observed increased fatal disease in inoculated younger rabbits and mice compared to their adult counterparts [5].

After several outbreaks in non-human primates from Asia over the next 10 years, increasing suspicion for an animal reservoir prompted the WHO to survey over 1500 samples from non-human primates in South East Asia and Africa [7]. However, virus was not isolated and none of the animals had positive poxvirus serology.

#### The Democratic Republic of the Congo, 1970

It has been suggested that MPXV may have been infecting humans for several hundred years and potentially misdiagnosed as smallpox. It was first diagnosed in a 9-month-old boy from the DRC in 1970 during efforts to eliminate smallpox [8]. The child first developed a fever followed by a rash which progressed peripherally, and samples of crusts were positive for MPXV clade I. Although the boy recovered fully, he developed secondary nosocomial measles and died six days later. Additional cases of clade I have since been reported across the Congo Basin, including the DRC, Cameroon, the Central African Republic, Gabon, and the Republic of the Congo. Additional sporadic cases of clade I have been identified in South Sudan, which, based on phylogenetic studies and further supported by the ecological and political landscape of South Sudan at the time of the outbreak, suggested importation of MPXV rather than transmission from indigenous animals to humans [9, 10]. Upon the declaration of eradication of smallpox in 1980, the WHO recognized the importance of continued surveillance of mpox and supported ongoing investigations into the natural history of the disease [11]. This resulted in an active surveillance program that continued until 1986. In a case series of 282 patients with confirmed mpox in the DRC during 1980–1985, 85% of those infected were less than 10 years old, and there was an 11% case fatality rate, with most deaths occurring in persons aged 0–4 years [12]. This case series informed our current understanding of a more severe disease course with clade I. However, outbreaks outside of Africa have not shown similar high case fatality rates [13, 14]. These data were used to create a stochastic model of MPXV human transmission. The model predicted an increase in secondary cases when there was complete absence of smallpox-induced immunity but anticipated declining cases rates with each successive generation and eventual termination of transmission [15, 16]. Based on these models, it was concluded that endemic mpox would be highly improbable and as such, was not considered a major public health problem. These data were also used to support the end of smallpox vaccine campaigns in MPXV-endemic areas by the WHO Global Commission for the Certification of Smallpox Eradication [11, 16]. This signaled the end of supported active surveillance programs in 1986, after which data on incidence and geographical burden of disease in endemic regions has been limited and incomplete.

#### The United States, 2003

Since 1970, cases of clade II have been identified and are now endemic to Sierra Leone, Nigeria, Liberia, Côte d’Ivoire, and Ghana. Infections due to clade II are documented to be less severe with less human-to-human transmission. This clade was responsible for the 2003 US outbreak, the first mpox outbreak outside of Africa [13]. An investigation by the United States Centers for Disease Control and Prevention (CDC) discovered 71 confirmed or probable cases of mpox, all traced to contact with infected pet prairie dogs from an Illinois animal distributor. The prairie dogs had previously been cohoused at a Texas distribution center with Gambian giant rats and dormice infected with MPXV imported from Ghana. The CDC also identified infected rope squirrels, tree squirrels, Gambian giant rats, brushtail porcupines, dormice, and striped mice at the Texas animal distribution center; however, no human cases were tied to these animals [17]. The human cases all resulted from contact with the infected prairie dogs with no person-to-person transmission of disease. The type of exposure to the infected animal influenced both the incubation period and disease severity. For persons with noninvasive exposure (any contact with an infected animal via intact skin, including physical contact, cleaning the cage of an infected animal, or being near an infected animal), the incubation period was approximately 13 days, akin to that observed in prior larger outbreaks in the Congo Basin [12]. However, persons with complex exposures (contact with infected animals resulting in a break in the skin, such as bite or scratch) experienced shorter incubation periods and were more likely to have pronounced systemic symptoms (*p* = 0.041) or be hospitalized (*p* < 0.001) [14]. There were no deaths in the US outbreak.

#### Nigeria, 2017 to present

In September 2017, almost four decades after its last reported local case in 1978, an outbreak of mpox was identified in Bayelsa State, Nigeria. Since this time, 247 confirmed cases and an additional 578 suspected cases have been reported across Southern Nigeria, with the majority of confirmed cases in the states of Rivers, Bayelsa, Lagos, Delta, and Cross River [18]. Athough cases continue to occur today; the lack of resources to conduct robust surveillance programs contribute to presumed underreporting. While reported case numbers initially dropped in successive years, the reported annual incidence quadrupled in 2021, and there was a subsequent doubling of cases in April 2022. Genetic sequencing suggests there were multiple sources of introduction of the virus into the human population from rodents (such as rats, squirrels, dormice striped mice) and non-human primates (including monkeys and chimpanzees) [19, 20]. Most infected persons have been men aged 20–40 years, with limited secondary person-to-person transmission. Waning *Orthopoxvirus*-related herd immunity from prior smallpox vaccination and increased human contact with animals related to deforestation, climate change, and farming and agriculture have been suggested as drivers for continued cases [21]. Given the rise in cases and the onset of the 2022 outbreak in non-endemic countries, the Nigerian CDC has since re-activated their Monkeypox Emergency Operations Center.

#### Worldwide outbreak, 2022

On May 6, 2022, a United Kingdom national returning from Nigeria was diagnosed with mpox after developing a febrile rash. This person would become the incident case in the 2022 outbreak of mpox in nonendemic countries worldwide. Up to 99% of all cases have been reported in gay, bisexual, and other men who have sex with men. On July 23, 2022, the WHO declared this outbreak a public health emergency of international concern [22]. There have now been over 78,000 cases reported from 109 locations [23]. All cases to date have been of clade III of MPXV but with a higher number of single-nucleotide polymorphisms (a mean of 50) than expected based on previous estimates of substitution rates (1–2 per genome per year) [24, 25]. Further genomic sequencing investigations are ongoing which will elucidate the possibility of accelerated viral evolution in humans, and may inform several unique features of this outbreak.

### Review of transmission, incubation period, and clinical characteristics

#### Transmission

In endemic countries, zoonotic transmission to humans was the primary mode of transmission, and person-to-person transmission was less common. Zoonotic transmission occurs directly through bites or scratches from infected animals or contact of lesions of infected animals with non-intact skin or mucous membranes. Indirect zoonotic transmission occurs from handling or cleaning cages of infected animals. Person-to-person transmission is documented with prolonged close contact with infected lesions, large respiratory droplets, or rarely vertical transmission (both transplacental and peripartum) [12, 26]. Fomite transmission is infrequent, and occurs through shared infected items such as linens and bedding [27]. Respiratory droplet transmission has been thought to be less efficient and only occurring with prolonged exposure of household or other close contacts. Airborne transmission is theoretical by extrapolation from observations with smallpox but no studies have assessed airborne transmission of MPXV. Transmission to healthcare workers in endemic countries is rare but has been described in cases with inappropriate personal protective equipment or with inadequate reprocessing of reusable medical devices, such as needles [28, 29]. In nonendemic countries, only one case of mpox has been reported in a healthcare worker who, while donning an apron and gloves but without an N95 respirator, may have aerosolized MPXV while changing infected bedsheets [29].

In the 2022 outbreak, person-to-person close contact has replaced zoonotic transmission as the predominant route of transmission. In an international case series of 528 persons with rtPCR-confirmed mpox, 99% were men (527/528), 96% were gay, bisexual, and other men who have sex with men (509/528), and 95% had sexual close contact as the suspected route of transmission (504/528) [30]. In a second series of 508 persons with confirmed mpox in Madrid, Spain, 99% were men (503/508), 78% were gay, bisexual, and other men who have sex with men (397/508), and 84% reported condomless sex or sex with multiple partners in the 21 days prior to symptom onset (427/508) [31]. Prior condomless sex or sex with multiple partners was reported in 84.1% of cases by Martinez et al. [31] The suspected route of transmission was sexual close contact in 95% of cases by Thornhill et al. [30] This raised the possibility of transmission through sexual fluids which has not previously been documented. Given early evidence of MPXV rtPCR-positive semen specimens, further analysis is required to determine the presence of viable MPXV in sexual fluids, and then whether this is sufficient to transmit disease [32]. Only a minority of cases reported contact with a person with confirmed mpox (26% and 5% as reported by Thornhill et al. and Martinez et al., respectively) [30, 31]. This may be due to the nature of sexual contacts being anonymous or may suggest the possibility of pre-symptomatic or asymptomatic transmission. Fomite transmission has not been reported in this outbreak. Transmission from an exclusive airborne route has also not been implicated. Research to inform the exact modes and timing of peak transmission is needed and ongoing.

#### Incubation period

In the early descriptions of people with mpox, the period between exposure to symptom onset, or incubation period, was approximately 13 days (range 5–21 days) [12]. However, exact timing was limited by the observational nature of data and recall bias inherent to diagnoses made retrospectively. On assessment of 29 cases from the 2003 outbreak in the United States, the time from animal exposure to symptom onset was 12 days (interquartile range, 11–28 days) [33]. More invasive exposures were associated with shorter incubation periods (9 days) compared to non-invasive exposures (13 days) [14]. However, these results, like those from the 1980s, reflect an incubation period after zoonotic exposure and are likely impacted by quantity of viral exposure and may not reflect the situation with human-to-human transmission. Classically, the period of infectivity is defined from onset of symptoms until all lesions have desquamated and re-epithelialized. This period typically lasts two to four weeks after the onset of the rash [12]. Transmission was not formerly known to occur in the incubation period.

In the 2022 outbreak, the observational nature of all current published cases precludes clear timelines of exposures and symptom onset. Furthermore, the nature of their exposures, namely multiple anonymous sexual contacts, often precludes accurate identification of transmission dates [31]. A clear chronological history was available for 30 of 528 cases by Thornhill et al., of which 23 had a clearly defined exposure. Of these 23 cases, the median incubation period was 7 days, ranging from 3 to 20 days [30]. Of 18 cases in the Netherlands during May 2022, the mean incubation period was 8.5 days (5th and 95th percentiles were 4.2 and 17.3 days, respectively) [34].

#### Clinical presentation

An observational cohort study identified 282 persons with confirmed mpox in the DRC during 1980–1985 [12]. 85% of cases were less than 10 years old; only 19 cases were 20 years of age and older. The disease began with a febrile prodrome of fever, chills, malaise, myalgias, back ache, and headache, lasting 8–12 days, and occurring 1–3 days before the onset of a vesiculopustular rash. Tender regional lymphadenopathy, if present, would occur before the onset of the first lesions and was an important clue to differentiate mpox from smallpox and chickenpox. The rash typically began on the face and trunk with centrifugal spread towards the palms and soles. Mucous membrane involvement was observed. Oropharyngeal lesions presented as a cough or pharyngitis. Lesions typically progressed through the stages of macules, papules, vesicles, and then pustules that umbilicated, ulcerated, scabbed over, then desquamated, leaving re-epithelialized, or healed, skin, a process that occurred over two to four weeks. Lesions were usually 5 mm in diameter, but occasionally up to 10 mm, and may be hemorrhagic, which made them distinct from the lesions of smallpox. Lesions were pruritic and/or tender. All lesions in a particular body region would evolve simultaneously in a process termed regional monomorphism, which contrasted to the regional pleomorphism of chickenpox. Over a period of weeks, the healed lesions appeared hypopigmented, and over the next several months, they become hyperpigmented as the final state.

The 2003 outbreak of infections due to MPXV clade II in the US provided additional objective information regarding the clinical presentation. Predominant signs and symptoms included rash (97%), fever (85%), chills (71%), lymphadenopathy (71%), headache (65%), and myalgias (56%) [33]. The fever lasted a median of 8 days (range 2–13 days), preceded the rash by a median of 2 days (range 0–12 days), and the rash lasted a median of 12 days (range 7–24 days). Mild laboratory abnormalities were noted, including leukocytosis, thrombocytopenia, elevated transaminases, and hypoalbuminemia. There were no deaths with supportive management.

#### Severe disease

The WHO developed a severity scale based on the number of lesions; 5–25 lesions was labeled benign, 26–100 was moderate, 101–250 was grave, and more than 250 was “plus grave” [8, 33]. Hospitalization due mpox or its complications or death due to mpox have also been classified as severe disease. In the DRC between 1980 and 1985 risk factors for severe disease included extremes of age, immunocompromising conditions including HIV, and pregnancy. Lack of smallpox vaccination was associated with a higher lesion count (*p* < 0.001) [12]. 78% of total deaths occurred in children less than 4 years old, highlighting young age as a risk factor [12]. A higher lesion count was associated with higher degree of fever, increased symptom severity, and longer duration of illness [12].

Several differences were noted in the 2003 US outbreak [33]. Vaccination status did not affect rate of severe disease, as defined by lesion count over 100 (*p* = 1.00), admission to hospital (*p* = 1.00) or admission to ICU (*p* = 1.00). There were no deaths in the 2003 US outbreak. While children were more likely hospitalized in an intensive care unit (*p* = 0.02), they were not more likely to develop severe disease (defined as fever over 38.3 °C [*p* = 0.70] and over 100 lesions [*p* = 0.32]) compared to adults. Nausea and/or vomiting (*p* = 0.005) were associated with a hospital length of stay over 48 h. On multivariate analyses, there were no significant risk factors associated with severe disease.

#### Complications

In the retrospective cohort study of 282 patients with mpox by Jezek et al. in the 1980s, 17% had secondary bacterial infections of cutaneous lesions, 11% had bronchopneumonia, 6% had GI symptoms, dehydration, and/or marasmus, 4% had keratitis or corneal ulcers, and 0.4% (1 patient) had encephalitis and all complications were more likely in those without previous smallpox vaccination [12]. Other common complications included acute tonsillitis and pharyngitis [12]. In 2003, there was also one case each of retropharyngeal abscess and epiglottitis. Other complications include sepsis and miscarriages[33].

#### Subclinical disease

Subclinical or inapparent disease has been defined as virologic or serologic evidence of infection without clinically apparent signs or symptoms of disease [8]. The first evidence of such was reflected in the recovery of MPXV from renal tissue of apparently healthy Cynomolgus monkeys from a colony with no cases of clinical monkeypox [8]. Subsequent investigation of 2510 human contacts of 214 cases of mpox between 1980 and 1984 found 13/449 (2.9%) of contacts unvaccinated against smallpox had positive mpox serology without past or present signs or symptoms of mpox [35]. This data was based on laboratory investigations of 449 of 641 total unvaccinated contacts. As contacts vaccinated against smallpox underwent laboratory investigation only when they showed signs or symptoms of disease (110 of 1869 vaccinated contacts), the lack of data on asymptomatic vaccinated contacts limited a true assessment of subclinical disease, especially stratified by vaccination status. This study also highlighted the difficulty in retrospectively distinguishing true subclinical disease, mild disease with no permanent scarring, and clinical disease without rash (akin to variola sine eruption with smallpox) in those with positive mpox serology. This study provided important qualitative evidence for the concept of subclinical disease, but additional research to quantify, identify risk factors, determine infectivity and long-term consequences of subclinical disease is needed. Serologic assays will need to distinguish between vaccine-mediated and natural immunity.

#### Impact of smallpox vaccination status on clinical disease

In the cases observed in the DRC between 1980 and 1985, prior smallpox vaccination was associated with attenuated disease, including fewer and smaller lesions (*p* < 0.001) and lower likelihood of lesions on the face (59% in vaccinated versus 97% in unvaccinated, *p* < 0.001), palms (38% in vaccinated versus 82% in unvaccinated, *p* < 0.001), and soles (31% in vaccinated versus 70% in unvaccinated, *p* < 0.001) [12]. In vaccinated patients, lesions were less likely on the genitalia (27% versus 10%), half as likely to occur in the oral mucosa, more likely to display regional pleomorphism (31% versus 18%) and less likely to display confluence (0% vs. 7%). Complications were much less frequent in vaccinated patients (9% versus 43%). As only 11% of patients in this cohort had evidence of prior smallpox vaccination, this is suggestive that smallpox vaccination had protective benefits against overall disease acquisition [12]. Similarly, a study of 2510 contacts of 214 patients with mpox demonstrated a secondary attack rate of 0.9% for vaccinated contacts compared to 7.2% for unvaccinated contacts [35].

National smallpox vaccination campaigns in the DRC ended in 1980. Twenty-five years later, an active surveillance program aimed at reassessing mpox in central DRC revealed an increased incidence, up to 20-fold in a single health zone, in the period from 2005 to 2007 compared to 1981–1986 [36]. This increased incidence was seen across all age groups. Only 3.8% of cases had evidence of prior smallpox vaccination, compared to 26.4% smallpox vaccination prevalence of the general population. 92% of cases were born after 1980, and a higher proportion of cases were 15 years of age or older (29% in 2005–2007 versus 8% in 1980–1985). This provided a signal for increasing incidence of mpox as smallpox immunity declined. However, causation has not been shown and the role of other factors, such as viral evolution towards increasing fitness, deforestation, increasing contact with animals, was not elucidated.

In the United States, national smallpox vaccination ended in 1972. During the 2003 US outbreak, prior smallpox vaccination did not alter median duration of illness, disease severity, or need for hospitalization [33]. It is important to note that the 1980–1985 cohort included cases from the DRC where clade I is endemic, but the 2003 US case series were all of clade II.

Through early observations of cases in 2022, several key differences from the historical understanding of the course of disease have arisen. First, the rash may be observed to occur before, with, after, or without the febrile systemic illness [30]. Second, the initial lesions, akin to primary syphilis [37], appear to occur at sites of inoculation, rather than starting on the face and trunk and spreading in a classic centrifugal fashion to extremities. Third, and in line with lesions at primary sites of inoculation, most patients diagnosed have anogenital lesions (72–73%), whereas 55% have lesions on the trunk or limbs, 25–36% on the face and 10–25% on palms and soles [30, 31]. These presentations may mimic that of other diagnoses, including sexually transmitted infections. This may influence early health seeking behaviour and which may contribute to an ascertainment bias in the location of primary lesions. Of note, the case series published in 1987 reported genital lesions in only 27% of unvaccinated cases and 10% of vaccinated cases [12]. Fourth, proctitis, which accompanies anogenital lesions, is being reported more frequently, and was reported in up to 16% of patients [30, 31]. Fifth, lesions are more often displaying regional pleomorphism, where multiple stages of lesions are simultaneously present on the same anatomical area. Sixth, lesions may also be smaller than classically described, including pinpoint, which are made prominent only due to surrounding erythema. It is unclear whether this may be due to a relatively smaller size of inoculation, greater host immunity, or lower virulence. Seventh, there appeared to be no increased risk of complications or hospitalization in those living with well-controlled HIV compared to those without HIV; however, this will require further study. Eighth, there have been several atypical presentations including myocarditis, ocular involvement and epiglottitis [30, 38]. Lastly, the case fatality rate is very low, 28/77,301 (< 0.001%) in countries not previously known to be endemic and 13/928 (0.014%) in countries traditionally known to be endemic [18]. However, this data is likely skewed by low-resource settings with differential access to healthcare, diagnostics, therapeutics, and vaccines.

### Review of diagnostic methods

A diagnosis of mpox is suspected based on clinical presentation in the right epidemiological context. However, given the similarities to other illnesses that include a fever and a vesiculopustular rash of public health concern, laboratory confirmation is required for diagnosis [39].

Histology and electron microscopy (EM) were previously used as an initial and rapid diagnostic test for mpox. Histology may reveal ballooning degeneration of basal keratinocytes and spongiosis of mildly acanthotic epidermis and, as lesions progress, may show progression to full thickness necrosis of marked acanthotic epidermis with few viable keratinocytes [40]. EM of lesion fluid and scabs reveal brick-like virions with lateral bodies and a dumbbell-shaped central core; it can differentiate an *Orthopoxvirus* from herpes simplex virus but require a skilled technician at a laboratory capable of EM. However, neither histology nor EM can distinguish between different *Orthopoxviruses*, and histology alone cannot distinguish MPXV from herpes simplex virus or varicella [40]. Along with histology and EM, viral culture was previously done for the detection of MPXV in prior outbreaks which produce distinctive pocks on chicken embryo chorioallantoic membranes and tissue [12, 13, 35]. Lesions were more reliable than blood since the viremic stage is shorter than viral shedding from lesions [41]. However, viral culture required a skilled technician and multiple days to complete and bacterial contamination can interfere with results. Similarly, immunohistochemistry, which had been used to test for the presence of *Orthopoxvirus*-specific antigens, required a skilled technician and could not differentiate MPXV [12, 35, 41]. In cases detected in the convalescent stage where lesions were unavailable, diagnosis was made retrospectively by *Orthopoxvirus* serology [12, 35]. Anti-*Orthopoxvirus* IgG was detected in patients with prior exposure to an *Orthopoxvirus* or to smallpox vaccine. Anti-*Orthopoxvirus* IgM was used to assess for recent immune response to an *Orthopoxvirus*, especially in those with remote smallpox vaccination; it may also indicate recent smallpox vaccination. Serology was cumbersome in that it required a cold chain and a skilled technician. The disadvantage to *Orthopoxvirus* serology was that it was nonspecific to MPXV as *Orthopoxviruses* are serologically cross-reactive.

In the 2003 US outbreak, diagnoses were made by a variety of methods including EM, immunohistochemical analysis, and/or viral culture [13, 33]. Real-time polymerase chain reaction (rtPCR) assay for *Orthopoxviruses* in general and for MPXV specifically were also used in many cases to make the diagnosis. Lesion fluid swabs, dry crusts, or biopsies were also preferred over blood given the relatively shorter viremic phase. Although rtPCR was known to be highly sensitive, contamination contributed to concern over false positive rates.

rtPCR assays have become increasingly available and are now the predominant method for definitive diagnosis of mpox in the 2022 outbreak [42]. rtPCR has the benefit of relatively earlier detection of disease and can be followed by restriction fragment-length polymorphism analysis or sequencing. Further investigation into performance characteristics will inform the optimal sampling sites. In the international case series, most samples were swabs of skin or genital lesions; throat and nasopharyngeal swab specimens and blood were less commonly tested [30]. The relative utility of pharyngeal or nasopharyngeal swabs, rectal swabs, urine, serum, and semen in cases without skin lesions also require further investigation [30–32]. Serology is currently unavailable widely. However, serology, specifically anti-*Orthopoxvirus* antibodies, are critical to surveillance, estimating the proportion of infections which may be asymptomatic, as well as monitoring for immunologic response (anti-neutralizing antibody titers). They may also be useful in the retrospective diagnosis of mpox for patients who are unvaccinated against smallpox.

### Review of differential diagnoses

Prominent lymphadenopathy and milder disease may suggest mpox but cases can be clinically indistinguishable from smallpox and thus a laboratory diagnosis is required. Given the global eradication of smallpox, mpox is much more likely. Other differentials for a vesiculopustular rash in the present day include infections caused by varicella-zoster virus, herpes simplex virus, *Treponema pallidum*, coxsackievirus A and other enterovirus serotypes, and molluscum contagiosum virus [43]. The rash of chickenpox generally occurs in successive crops of lesions and display regional pleomorphism; however, this cannot be used to differentiate mpox in the 2022 outbreak. Thus, an epidemiological history compatible with chickenpox may be confirmed with a varicella-zoster virus PCR of a lesion swab to definitively rule out mpox. The rash of herpes zoster can be distinguished from mpox by its classically dermatomal distribution. Primary syphilis presents as a painless solitary chancre at the site of inoculation after unprotected sex, compared to mpox which may start as a primary solitary lesion but typically progresses to include additional lesions on multiple parts of the body. The rash of secondary syphilis may occur throughout the body including the trunk, extremities, palms, soles, and oral mucosal membranes, may become confluent around intertriginous areas and can be distinguished by treponemal and nontreponemal tests. Hand, foot and mouth disease can be distinguished based on its classic presentation of lesions limited to the hand, feet, and mouth with a compatible epidemiological link (such as outbreaks within children or contact with another case of hand, foot, and mouth disease). Molluscum contagiosum may be distinguished based on its classic appearance of dome-shaped and flesh-colored papules with central umbilication that are rarely painful and can occur anywhere on the body but are uncommon in the mouth, or on palms or soles.

### Review of management

The management of mpox is largely supportive and focused on infection control. Two agents have been proposed to have efficacy in mpox: brincidofovir and tecovirimat. PALM 007, a randomized, placebo-controlled, double-blinded trial to assess the safety and efficacy of tecovirimat for the treatment of mpox in the DRC and supported by the WHO, is underway, as is the CORE PROTOCOL, an international, randomized, placebo-controlled adaptive platform trial to assess the same and other treatments for patients with mpox. Randomized controlled trials are also initiated in the UK (PLATINUM trial), Canada (PLATINUM-CAN), and the US (STOMP) to assess efficacy of antiviral treatments.

#### Tecovirimat

Tecovirimat (formerly, ST-246; brand name, TPOXX^®^) was the first antiviral medication approved for the treatment of smallpox by the FDA in 2018, Health Canada in 2021, and the European Medicines Agency (EMA) in January 2022. For mpox, the EMA is the only federal agency to have approved tecovirimat for the treatment of mpox. In the US and Canada, tecovirimat is currently available for mpox only through government strategic national stockpiles or randomized controlled trials. Tecovirimat is currently indicated as emergency release in cases of mpox with severe disease or those with risk factors for severe disease, including immunocompromising conditions, pregnancy, breastfeeding, pediatrics atopic dermatitis, and severe complications of mpox.

Tecovirimat inhibits a highly conserved *Orthopoxvirus* VP37 envelope protein that is thought to contribute to transmission between cells and through the bloodstream to cause disseminated disease [44, 45]. To date, reports of tecovirimat resistance is infrequent, but could emerge as resistance can result from a single amino acid mutation [45, 46]. A case of resistance occurred near the end of a 73-day treatment course complicated by periods of subtherapeutic levels and concomitant topical drug application in a patient with underlying immunocompromise and progressive vaccinia [47]. As a drug patented in 2004, investigating its safety and efficacy in humans for treatment of smallpox was not possible given the previous eradication of smallpox. Thus, the approval of tecovirimat for smallpox was based on the “Animal Rule”, a regulation allowing timely approval of drugs for serious conditions using animal models when studies in humans would be unethical or not possible [48]. In studies of non-human primates inoculated with lethal doses of MPXV, tecovirimat significantly reduced risk of death, viral load, and lesion counts, especially when given earlier in disease course [49]. However, tecovirimat was found to be less effective in immunocompromised animal models [50, 51]. Safety and tolerability of tecovirimat has been shown in phase I and II placebo-controlled trials of 449 healthy adults [49]. Tecovirimat has been used infrequently for the treatment of mpox [52, 53]. In a small case series of persons with mpox in the UK between 2018 and 2021, one patient received tecovirimat and had no adverse effects, shorter duration of viral shedding and illness compared to six other patients [53].

Tecovirimat is prescribed as 600 mg oral twice daily for a 14-day course. The most common side effects include headache, abdominal pain, nausea, and vomiting. There is no safety or efficacy data for children or adolescents less than 17 years old, for persons who are pregnant or breastfeeding, or persons over the age of 65 years. Tecovirimat is not contraindicated in pregnancy or breastfeeding, but its use should follow a risk-benefit discussion and shared decision making. The product monograph lists no contraindications to the use of oral tecovirimat [46]. However, there are important drug-drug interactions. Tecovirimat with repaglinide causes hypoglycemia, and tecovirimat can decrease the effectiveness of midazolam. With earlier versions of the smallpox vaccine, vaccine effectiveness for smallpox was reduced when given with tecovirimat; the global eradication of smallpox has precluded an assessment of this effect with the third-generation vaccines but is hypothesized to not occur [54]. In one study of mice inoculated with cowpox, tecovirimat co-administered with brincidofovir had greater mortality benefit compared to either drug alone, especially when therapy was delayed, with no increase in toxicity [55]. Clinical trials are in development to study tecovirimat in several jurisdictions.

#### Brincidofovir

Brincidofovir (formerly, CMX001; also referred to as hexadecyloxypropyl-cidofovir; brand name Tembexa^®^) was approved for the treatment of smallpox by the FDA in 2021; no other federal agency has approved its use in smallpox, and no federal agency has approved its use for the treatment of any other disease, including mpox [56]. In the US, brincidofovir is currently unavailable from the strategic national stockpile, but the CDC is developing an expanded access investigational new drug protocol for use with mpox.

Brincidofovir is a long-acting prodrug of cidofovir lipid-conjugated to achieve superior oral bioavailability. It is readily converted to cidofovir intracellularly to inhibit *Orthopoxvirus* DNA polymerase–mediated viral DNA synthesis. Compared to cidofovir, it achieves higher concentrations in lung, spleen, and liver [57]. It achieves lower renal concentrations, decreasing the risk of nephrotoxicity [58]. In murine and rabbit models of smallpox, brincidofovir had a mortality benefit, particularly when given early in disease [59, 60]. In a placebo-controlled, double-blind phase III trial for use as cytomegalovirus (CMV) prophylaxis in immunocompromised adults, brincidofovir was not helpful in reducing CMV infection, was associated with more adverse events, and with prolonged use (24 weeks), slightly higher all-cause mortality [61]. This forms the basis for the US boxed warning of increased mortality with prolonged use. Similar to tecovirimat, brincidofovir was approved for smallpox based on the “Animal Rule”, and there are no human randomized controlled trials exploring the efficacy of brincidofovir in mpox. Its use was evaluated in a small case series of humans in the UK between 2018 and 2021, demonstrating, in 3 patients, a transient reduction in MPXV PCR cycle threshold that was neither durable nor consistent between patients and associated with elevated liver function tests [53].

Oral brincidofovir is favoured over the use of intravenous therapy, which should be reserved when the oral route is not possible. The most common side effects include mild gastrointestinal symptoms (abdominal pain, nausea, vomiting, and diarrhea) and asymptomatic, transient, and reversible elevations in serum transaminases [62]. There are no contraindications to brincidofovir. Co-administration of brincidofovir and inhibitors of OATP1B1 and 1B3 (such as clarithromycin, erythromycin, rifampin, cyclosporine, gemfibrozil, HIV protease inhibitors) may increase the risk of brincidofovir-associated adverse reactions [56]. There are no safety or efficacy data in pregnancy or breastfeeding. Fetal harm from brincidofovir was suggested by animal reproduction studies [56]. Given the risk of smallpox transmission through breastmilk, the recommendation to avoid breastfeeding during active infection precludes evaluation of brincidofovir in this context. Furthermore, brincidofovir is considered a potential human carcinogen and may have irreversible testicular toxicity causing infertility [56].

### Smallpox vaccination 

There is no vaccine specific to mpox and no human studies were found at the time of our review assessing the vaccine effectiveness of any smallpox vaccine for any indication in the 2022 mpox outbreak. Furthermore, no human studies were found assessing antibody titer required to prevent mpox. However, animal studies suggested smallpox vaccination reduced the risk of disease due to MPXV and attenuated disease severity [63–65]. There are two available smallpox vaccines: ACAM2000^®^ and Modified Vaccinia Ankara-Bavarian Nordic. Pubic health departments have been releasing these vaccines for compassionate use.

#### ACAM2000^®^

ACAM2000^®^ is a second-generation smallpox vaccine that contains live, replication-competent vaccinia virus that replaced its predecessor, Dryvax [66]. ACAM2000^®^ was licensed by the FDA in 2007 for prevention of smallpox in high-risk populations and is stored in the US Strategic National Stockpile [63]. In the US, it can be used through an expanded access investigational new drug protocol. It has not been approved by the EMA or Health Canada. Its efficacy for smallpox prevention has been suggested by human and animal trials, including immunologic studies of humans that showed noninferiority to Dryvax [63–65]. No studies were found assessing its efficacy for prevention of mpox.

The primary series of ACAM2000^®^ consists of a single percutaneous dose administered via a multi-puncture inoculation technique with a bifurcated needle [63]. A cutaneous reaction, or “take”, develops at the site of inoculation and heals over 6 weeks; it is classically used as a biomarker of immune response. Immunity is conferred at 28 days. In a trial of ACAM2000^®^ assessing its safety, common local adverse events included pruritis (up to 92%), pain (up to 77%), erythema (up to 74%) and swelling (up to 48%) at the injection site [63, 65]. Other common adverse events included lymph node pain (up to 57%), headache (up to 50%), fatigue (up to 77%), malaise (up to 60%), myalgia (up to 60%), and subjective fever (up to 37%), and gastrointestinal symptoms (up to 31%) [63, 65]. The “take” risked inadvertent autoinoculation when left uncovered (529.2 cases per million primary vaccinations), and most commonly occurred to the face including eyes and lips, genitalia, and rectum. Serious adverse events were rare and included myocarditis or pericarditis (occurring in 5.7 per 1000 primary vaccinations), encephalitis (12.3 per million primary vaccinations), and progressive vaccinia (1.5 per million primary vaccinations) [67–69]. People who had atopic dermatitis or other eczematous conditions were at higher risk of developing eczema vaccinatum. ACAM2000^®^ may result in a false-positive RPR test and thus a positive RPR should be confirmed by a treponemal test. ACAM2000^®^ may also cause a false-negative tuberculin skin test and as such, tuberculin testing should be delayed for 1 month after smallpox vaccination.

Given that it is a live vaccine, the vaccine is contraindicated in those with severe immunodeficiency. The theoretical risk of fetal infection and death has precluded any studies during pregnancy or lactation, and so ACAM2000^®^ is listed as a pregnancy category D. There were no trials assessing the safety and efficacy of ACAM2000® for people of age of 16 years and younger; however, its use in this age group is supported by evidence in adult populations and evidence from prior vaccines that were routinely administered to all pediatric groups including neonates and infants before the global eradication of smallpox. There were no trials assessing the safety and efficacy of ACAM2000^®^ in persons over the age of 65 years.

#### Modified Vaccinia Ankara-Bavarian nordic

Modified Vaccinia Ankara-Bavarian Nordic (MVA-BN; trade names, Imvamune^®^, Imvanex^®^, Jynneos™) is a third-generation smallpox vaccine that contains a live but non-replicating form of vaccinia virus. MVA-BN was approved for prevention of smallpox and related *Orthopoxvirus* infections including mpox in persons aged 18 years and older who are at high risk of infection in the European Union in 2013 (under the trade name Imvanex^®^), by the US FDA in 2019 (under the trade name Jynneos™), and by Health Canada in 2020 (under the trade name Imvamune^®^) [70, 71].

Like ACAM2000^®^, there were no clinical trials assessing the efficacy of MVA-BN for smallpox as it had already been eradicated. In a phase 3, open-label, randomized noninferiority trial, peak serum neutralizing antibody titers for MVA-BN were noninferior to that of ACAM2000 [72]. MVA-BN also resulted in significant “take” attenuation after subsequent administration of ACAM2000, which was used to demonstrate effectiveness [72]. There were no studies assessing the safety or efficacy of MVA-BN in mpox. Vaccine effectiveness for mpox is suggested based on data for smallpox. There were no studies assessing the safety or efficacy of MVA-BN in persons less than 18 years of age or those with immunocompromising conditions or taking immunosuppressant medications. There were no studies assessing safety and efficacy in pregnancy or safety with breastfeeding. As this vaccine is nonreplicating in human cells, its administration is supported in these populations. It is unknown whether childhood immunization would be effective now with the 2022 outbreak.

A primary series consists of 2 subcutaneous injections 28 days apart. Immunity is conferred 14 days after the second dose. As MVA-BN is non-replicating, no scar is left behind. In a study of 212 people, local adverse reactions include pain (52%), erythema (36%), swelling (16%), induration (15%), and pruritis (17%) at the site of injection [72]. Systemic adverse reactions included fatigue (25%), headache (25%), myalgias (24%), and nausea (9%) [72]. This vaccine is contraindicated in people who have had previous serious hypersensitivity to any component of the formulation. There are trace amounts of ciprofloxacin and gentamicin in MVA-BN that presents a small but present risk of allergic reaction to the vaccine. This risk of myocarditis or pericarditis is unknown for MVA-BN, but possible based on extrapolation of such a risk with ACAM2000^®^. Given the independent risks of myocarditis and pericarditis after either mRNA COVID-19 vaccine or ACAM2000®, there is a theoretical increased risk of cardiac adverse events when the two vaccines are co-administered. Combining the results of 7414 persons in 20 studies, serious cardiac adverse events were documented to occur in 1.4% of persons who received Imvamune^®^ compared to 0.2% of persons who received a placebo vaccine [73]. There were no cases of cardiac inflammatory disease. While one may consider separating MVA-BN and mRNA COVID-19 vaccines, this is not currently recommended by the CDC during the 2022 mpox outbreak [18]. While decreased immune response to ACAM2000^®^ with co-administration of tecovirimat or brincidofovir has been observed, it is unknown if this can occur with MVA-BN.

In the 2022 outbreak, the CDC has recommended the use of smallpox vaccine for post-exposure prophylaxis and pre-exposure prophylaxis [74]. Post-exposure prophylaxis is recommended within 4 days of exposure for optimal benefit, although it can be given within 14 days to reduce symptoms of disease. However, vaccination after the onset of signs or symptoms may not alter the clinical course and so this is not supported in the context of limited vaccine supply. Preexposure prophylaxis to people at increased risk of exposure to MPXV is also supported. Based on current observations, those at highest risk include people with male genitalia including gay, bisexual, and other men who have sex with men, transgender woman, gender non-conforming or gender non-binary people who are similarly at increased risk, especially those who have had multiple or anonymous recent sexual encounters. The CDC terms this use as “expanded postexposure prophylaxis” to acknowledge those who may have had unknown recent exposures to a person with mpox [74]. Smallpox vaccination as preexposure prophylaxis is also recommended for laboratory workers who work with MPXV.

## Discussion

MPXV has been endemic in several countries since the 1970s with a classically described zoonotic transmission and a clinical presentation that included a febrile prodrome followed by a rash that progresses monomorphically over 2–4 weeks. Observations from the 2022 outbreak in nonendemic countries across the world have noted changes in the epidemiology and clinical presentation of mpox. Most cases in the 2022 outbreak have been in gay, bisexual, and other men who have sex with men, transgender woman, gender non-conforming and gender non-binary people who have had unprotected sex or multiple or anonymous sexual partners. In the 2022 outbreak, the rash commonly starts in the anogenital and oropharyngeal regions and does not reliably begin after a febrile prodrome. Despite our knowledge of mpox and its outbreak potential for decades, there has been little research into antivirals or vaccines. This was likely because the infection was thought to be an uncommon occurrence, although lack of surveillance programs precluded an accurate understanding of disease incidence. As well, cases were primarily in resource-poor settings, limiting dedicated research into therapeutics and vaccines.

There is no randomized controlled data available to support the use of anti-virals like tecovirimat, although it is available in many countries as an emergency release agent for severe cases, and the definitive trials are ongoing. Currently, there is also limited supply of smallpox vaccines and no data to support vaccine effectiveness. Until a larger supply can be obtained and data available, public health units are attempting an equitable approach to vaccine access and distribution. This includes a nonjudgmental approach to prioritizing populations at highest risk of severe disease and death, leveraging a variety of stakeholders, and engaging people from within affected communities to serve as trusted healthcare advocates. Administration of one dose of the two-dose MVA-BN vaccine may serve to provide more people with adequate protection against severe disease and death; this is the current recommendation of Canada’s National Advisory Committee on Immunization. Use of lower volumes through intradermal administration is also being used in the United states. Transmission to healthcare workers has mostly occurred in places where MPXV is endemic and where suboptimal infection prevention and control measures have been implicated [28]. Healthcare transmission in non-endemic areas with appropriate use of personal protective equipment has not occurred [29]. The risk to healthcare workers and other communities outside of those at highest risk is low and as such vaccination as preexposure prophylaxis for these groups is not recommended.

## Conclusion

There are many features of the 2022 human monkeypox outbreak that are different from the historical endemic cases. Clinicians need a better understanding of the clinical presentation, risk factors for severe disease and complications to allow for optimal diagnosis and care for people with mpox. Research is needed to gain a clear understanding of how genetic mutations may affect phenotypic disease, affect the molecular methods used to detect MPXV and diagnose disease, and affect the potential for antiviral resistance. For public health agencies to provide clear guidance on case and contact management and infection prevention and control, clearly establishing the modes of transmission and the periods of incubation and transmissibility are essential. Public health agencies would also benefit from better understanding the potential for reverse zoonosis and its role in establishing an animal reservoir in nonendemic countries. Determining the safety and efficacy of vaccines and antivirals in preventing and modulating disease through randomized controlled clinical trials using a collaborative global approach is needed and ongoing.

## Data Availability

Data sharing is not applicable to this article as no datasets were generated or analysed during the current study.

## Abbreviations

MPXV: Monkeypox virus
DRC: Democratic Republic of the Congo
WHO: World Health Organization
CDC: Centers for Disease Control and Prevention
EM: Electron microscopy
VZV: Varicella-zoster virus
FDA: Food and Drug Administration
EMA: European Medicines Agency
MVA-BN: Modified Vaccinia Ankara-Bavarian Nordic
